# Postpartum Uterine Dehiscence and Rupture Following Successful Vaginal Birth After Cesarean: A Case Series

**DOI:** 10.7759/cureus.109996

**Published:** 2026-05-31

**Authors:** Nika Bakshi, Gayaneh Nazarian, Stephanie Wong

**Affiliations:** 1 Obstetrics and Gynecology, Oakland University William Beaumont School of Medicine, Auburn Hills, USA; 2 Obstetrics and Gynecology, Beaumont Health, Royal Oak, USA

**Keywords:** hemoperitoneum, postpartum hemorrhage, trial of labor after cesarean section(tolac), uterine dehiscence, uterine rupture, vaginal birth after cesarean section

## Abstract

Uterine rupture is a rare and potentially fatal complication seen after vaginal birth after cesarean section (VBAC). We present two cases of uterine dehiscence and uterine rupture following successful VBAC, each demonstrating distinct clinical presentations and outcomes.

A 34-year-old gravida 2, para 1001 developed persistent brisk bleeding after a successful VBAC, raising concern for uterine dehiscence. She was found to have a 2-cm anterior uterine defect that was successfully repaired. In contrast, a 31-year-old gravida 2, para 1001 developed hemodynamic instability following a successful VBAC and was found to have a 6-cm uterine rupture of the anterior wall, contiguous with a 4-cm posterior defect. The patient was started on a massive transfusion protocol and initially stabilized in the operating room; however, due to recurrent hemodynamic instability, an abdominal hysterectomy was performed.

These cases highlight a rare yet serious complication of trial of labor after cesarean, with uterine dehiscence and rupture occurring at the prior hysterotomy site following successful VBAC. There is limited literature on the postpartum diagnosis of uterine rupture and dehiscence in the setting of VBAC; this report provides insight into their variable clinical presentations and management.

## Introduction

Uterine rupture is a potentially life-threatening complication for the mother and fetus. It is clinically defined as a full-thickness tear involving all uterine layers, including the serosa [1]. Uterine rupture is associated with significant maternal and fetal adverse outcomes, including maternal hemorrhage, coagulopathy, hemodynamic instability, fetal hypoxia, or maternal and/or fetal death. This is in contrast with uterine dehiscence, which is an incomplete tear or partial separation in which the serosa remains intact [1]. This can appear as a uterine window that may be seen at the time of cesarean section; however, uterine dehiscence typically is not associated with significant adverse perinatal outcomes.

Rupture most commonly occurs in patients with a previous uterine scar, with 90% of these cases involving a scar from a previous cesarean [2]. The estimated incidence of uterine rupture during a trial of labor after cesarean (TOLAC) is between 0.3% and 1.0% [1]. This rate is higher than the 0.03% incidence of rupture for those with planned cesarean sections [1]. While rupture rates during delivery are known, the incidence of rupture diagnosed following delivery remains largely unknown, with only two known cases reported in the literature [3,4].

Although less common, rupture of an unscarred, gravid uterus can also occur. These cases may be secondary to trauma following a motor vehicle collision or to obstetric maneuvers such as internal or external cephalic version [5,6]. They can also be seen in patients with congenital myometrial weakness, such as those with osteogenesis imperfecta type 1 or Ehlers-Danlos type IV [5,6]. Moreover, rupture of an unscarred uterus can occur due to the coexistence of various risk factors such as uterotonic agents, high parity, macrosomia, short interpregnancy interval, and advanced maternal age [5,6].

In this report, we present one case of uterine dehiscence and another case of uterine rupture following a successful vaginal birth after cesarean section (VBAC), highlighting the importance of recognition and prompt intervention to optimize maternal outcomes.

## Case presentation

Case 1

A 34-year-old gravida 2, para 1001 at 39 weeks and 3 days’ gestation presented to the hospital for a scheduled elective induction of labor following a low transverse cesarean for breech presentation two years prior, with an interpregnancy interval of 13 months. Of note, the operative report from the primary cesarean section described a thin lower myometrium, and the hysterotomy was closed with a one-layer running, locking stitch.

Oxytocin was used for the patient’s induction, and a cervical Foley catheter was placed for mechanical dilation. After nine hours of induction, the patient entered the second stage of labor. After one hour of pushing, the patient delivered a 3,555 g infant in a successful VBAC. Following a spontaneous placental delivery, increased vaginal bleeding was visualized. One dose of 0.2 mg of intramuscular methergine was given due to lower uterine atony. A small piece of placenta was manually extracted, and a second-degree perineal laceration was repaired. The cervix was visualized to be intact without laceration; however, the increased bleeding persisted. Differential considerations at that time included cervical laceration, uterine atony, retained products of conception, uterine rupture, and uterine dehiscence. Bedside ultrasound demonstrated a clean endometrial stripe, along with a collection of complex appearing fluid outside the uterine cavity, contiguous with the prior uterine hysterotomy. Manual evaluation of the uterine cavity revealed a 2-cm anterior uterine defect, and the patient was taken back for an exploratory laparotomy with concern for uterine rupture.

The patient was given preoperative antibiotics, and the prior Pfannenstiel skin incision was used for the laparotomy. Upon entering the peritoneal cavity, scant clear peritoneal fluid with no frank blood was noted. Significant edema of the serosal uterine layer was visualized, and a 3-cm dehiscence was palpated on the right side of the prior hysterotomy site. The dehiscence was incised, and the hysterotomy was closed with a one-layer running, locking stitch using 0-Vicryl. An additional dose of 0.2 mg of intramuscular methergine was given for lower uterine atony prior to fascial and skin closure. Postoperatively, the patient continued to have lower uterine atony, and an intrauterine suction device (JADA® System, Organon & Co., Jersey City, NJ, USA) was placed. When the patient became hypotensive with a hemoglobin of 8.4 g/dL, down from 13.9 g/dL on postoperative day 0, she was transfused with one unit of packed red blood cells. Otherwise, the patient’s postoperative course was uncomplicated, and the patient was ultimately discharged on postoperative day 2.

Case 2

A 31-year-old gravida 2, para 1001, at 40 weeks and 5 days’ gestation presented in early labor. Her obstetric history was notable for a primary low transverse cesarean section three years ago due to breech presentation, with an interpregnancy interval of 21 months. The operative report of the primary cesarean section noted a two-layer closure of the uterus with a running, locking stitch followed by an imbricating stitch.

Upon admission, the patient received penicillin for group B streptococcus positivity, and within 3 hours, the patient entered the second stage of labor. A vacuum-assisted delivery was performed due to nonreassuring fetal heart tones, resulting in the delivery of a healthy 3,550 g infant. After repair of the second-degree perineal laceration and right mediolateral episiotomy, continuous steady bleeding prompted an ultrasound evaluation, where a moderate clot burden was visualized in the lower uterine segment. A manual extraction of clots was performed; however, within the following hour, the patient became hypotensive, with blood pressure decreasing to 59/30 mmHg. Hemoglobin decreased from 11.2 g/dL to 7.7 g/dL, and the patient was transfused. Differential considerations included uterine atony, disseminated intravascular coagulation, uterine rupture, and retroperitoneal hematoma.

After a second manual extraction of clots, ultrasound demonstrated reaccumulation of a small clot burden in the lower uterine segment, with no free fluid in the vesicouterine or rectouterine pouches. Two attempts were made for accurate placement of the JADA® System; however, with continued bleeding around the device and a postpartum hemorrhage of 2.2 L, a decision was made to proceed with suction dilation and curettage, and a massive transfusion protocol was initiated. Additional uterotonics, including hemabate and methergine, were administered prior to transfer to the operating room.

In the operating room, another manual extraction of the clots was performed, noting continued lower uterine atony. Additionally, a 6-cm anterior defect was palpated and found to be contiguous with a 4-cm posterior defect, raising concerns for a uterine rupture. The patient underwent an exploratory laparotomy using the prior Pfannenstiel skin incision, where a large amount of hemoperitoneum was evacuated. A 6-cm full-thickness defect was appreciated in the right posterolateral uterine wall contiguous with the lower uterine segment at the prior hysterotomy site. The uterine rupture defect was repaired with running, locking stitches.

A large anterior hematoma was noted to extend across the lower uterine segment and into the repaired posterior uterine wall and the right broad ligament. The gynecology oncology team was consulted intraoperatively. Pressure was held at the hematoma, which remained stable without further expansion. Despite application of hemostatic agents, including Surgiflo® and Surgifoam® (Ethicon, Somerville, NJ, USA), bleeding was again noted at the repair site. The right utero-ovarian ligament was cauterized using the LigaSure™ device (Medtronic, Minneapolis, MN, USA) to decrease blood flow and blood loss. Due to continued slow bleeding, the patient was temporarily packed and transferred to the interventional radiology suite for bilateral uterine artery embolization (UAE).

Following UAE, the abdomen was re-explored with a midline vertical skin incision, and approximately 2 L of hemoperitoneum was evacuated. The anterior hematoma previously seen was stable and non-expanding. Slow bleeding was noted from the left mesosalpinx, which was then made hemostatic with the LigaSure™ device and suture. The patient was closed and transferred to the surgical intensive care unit (SICU).

Approximately two hours after arrival to the SICU, the patient became hypotensive, requiring increased vasopressor support. Due to increasing abdominal distension and vaginal bleeding, the patient returned to the operating room. Approximately 5-6 L of both clotted and free blood were evacuated. No significant findings were otherwise noted, and a total abdominal hysterectomy was performed with the assistance of a gynecology oncologist. Within 24 hours, the patient lost approximately 15 L of blood and was transfused with a total of 29 units of packed red blood cells (PRBCs), 16 units of fresh frozen plasma (FFP), 7 units of platelets, and 2 units of cryoprecipitate. The patient was then managed in the SICU for three days and ultimately discharged on postoperative day 6.

A comparison of the clinical presentation, diagnostic findings, and management strategies between the two cases is summarized in Table 1. Additional objective clinical data, including hemodynamic parameters, laboratory findings, and transfusion requirements, are summarized in Table 2.

**Table 1:** Comparison of clinical presentation, diagnostic findings, and management between two cases of postpartum uterine dehiscence and rupture following vaginal birth after cesarean.

Feature	Case 1 (Uterine Dehiscence)	Case 2 (Uterine Rupture)
Timing of diagnosis	Postpartum	Postpartum
Initial presentation	Persistent vaginal bleeding	Postpartum hemorrhage with hemodynamic instability
Intrapartum warning signs	Absent	Nonreassuring fetal heart tracing
Risk factors	One uterotonic agent used, short interpregnancy interval of 13 months	Two uterotonic agents used
Ultrasound findings	Fluid collection adjacent to uterine cavity	Clot burden in the lower uterine segment
Defect characteristics	2-3 cm of anterior uterine defect	6-cm anterior defect contiguous with 4-cm posterior defect
Management	Exploratory laparotomy with repair	Exploratory laparotomy with repair, uterine artery embolization, interval hysterectomy
Blood loss	456 mL at the time of delivery, followed by 340 mL during exploratory laparotomy	Approximately 15 L of total blood loss
Outcome	Uterine preservation	Massive transfusion protocol and hysterectomy
Differential diagnosis	Cervical laceration, uterine atony, retained products of conception, uterine rupture	Uterine atony, disseminated intravascular coagulation, uterine rupture, retroperitoneal hematoma

**Table 2:** Comparison of objective clinical data between postpartum uterine dehiscence and uterine rupture following vaginal birth after cesarean. PRBC, packed red blood cell, FFP, fresh frozen plasma

Clinical Variable	Case 1 (Uterine Dehiscence)	Case 2 (Uterine Rupture)
Prior uterine closure	One-layer locking closure	Two-layer closure
Labor characteristics	Induction with oxytocin and Foley balloon	Spontaneous labor with vacuum-assisted delivery
Lowest recorded blood pressure	Transient hypotension (80/53 mmHg)	Hemorrhagic shock shortly after delivery (59/30 mmHg)
Hemoglobin difference from initial admission	5.5 g/dL	3.5 g/dL
Transfusion requirement	1 unit of PRBCs	29 units of PRBCs, 16 units of FFP, 7 units of platelets, 2 units of cryoprecipitate

## Discussion

Understanding the varying clinical presentation of both uterine rupture and dehiscence is crucial for timely diagnosis and management. The classic clinical symptoms of uterine rupture depend greatly on when it occurs. Intrapartum ruptures typically present with acute signs such as abdominal pain, hemodynamic instability, fetal distress, and loss of fetal station [1,7]. Conversely, postpartum uterine rupture typically presents as persistent abdominal pain, vaginal bleeding refractory to uterotonics, and hematuria if the rupture extends into the bladder [1,7]. In contrast, uterine dehiscence may present asymptomatically with minimal bleeding or abdominal distension, as the serosal layer is intact [1].

In the first case, there was an increased concern for uterine dehiscence shortly after delivery due to continued vaginal bleeding. This patient lacked any intrapartum warning signs, such as abdominal pain, fetal distress, or hemodynamic instability. Instead, persistent vaginal bleeding prompted further evaluation, where ultrasound demonstrated findings concerning for uterine dehiscence, which was ultimately repaired, allowing for uterine preservation. Similar to a previously reported postpartum uterine dehiscence case, ultrasound findings in our first case aided in early suspicion for a uterine scar defect despite the absence of classic intrapartum signs [3]. This further highlights both the potential utility of ultrasound in the postpartum evaluation of suspected uterine scar complications and the importance of maintaining uterine rupture or dehiscence in the differential diagnosis when assessing postpartum hemorrhage.

The second case demonstrates a severe maternal outcome, in which the patient experienced significant hemodynamic instability and was found to have an extensive uterine rupture involving both anterior and posterior defects. Given the complex nature of this case, a multidisciplinary approach, which included the gynecologic oncology, intensive care, and interventional radiology teams, was used to stabilize the patient, who ultimately underwent an abdominal hysterectomy. This case highlights the need for surgical management of an extensive uterine rupture when conservative measures fail to achieve hemostasis.

While there is very limited literature on uterine rupture after successful VBAC, our two cases demonstrate significant variation in the presentation of uterine rupture and dehiscence in the postpartum setting. This is consistent with prior reports of postpartum uterine dehiscence presenting with persistent vaginal bleeding and delayed diagnosis following successful VBAC, as well as postpartum uterine rupture presenting with rapid hemodynamic deterioration and hemoperitoneum requiring emergent surgical intervention [3,4]. Aside from prior cesarean delivery and successful TOLAC/VBAC, there were no clearly consistent clinical risk factors identified across these cases, further emphasizing the importance of maintaining clinical suspicion for uterine scar complications even in patients without classic warning signs [3,4].

Due to the broad spectrum in presentation and severity of these injuries, the management of uterine rupture and dehiscence is determined by hemodynamic stability, the extent of the tear, and the patient’s desire for fertility [1]. Because these injuries can vary substantially in both location and extent of damage, an optimal surgical repair technique has yet to be established [1]. Moreover, there is no published evidence supporting routine manual examination of the uterine cavity or routine postpartum imaging of the cesarean scar. Further research is needed to better characterize postpartum presentations of uterine scar complications and optimize diagnostic evaluation in the postpartum setting.

## Conclusions

Understanding the presentation and potential outcomes of uterine rupture and dehiscence is important in reducing maternal morbidity and mortality. Our two cases demonstrate variable postpartum presentations of uterine rupture and dehiscence following successful VBAC, ranging from persistent vaginal bleeding to profound hemodynamic instability requiring hysterectomy. Although limited by the small number of cases, these findings highlight the importance of maintaining clinical vigilance for uterine scar complications in the postpartum period, particularly in patients with persistent bleeding or otherwise unexplained clinical deterioration despite initially nonspecific findings. While ultrasound findings were nonspecific in both cases, bedside ultrasound may still assist clinicians in narrowing the differential diagnosis and prompting further evaluation.
